# Cesium Lead Iodide Perovskites: Optically Active Crystal Phase Stability to Surface Engineering

**DOI:** 10.3390/mi13081318

**Published:** 2022-08-15

**Authors:** Yixi Wang, Hairong Zhao, Marek Piotrowski, Xiao Han, Zhongsheng Ge, Lizhuang Dong, Chengjie Wang, Sowjanya Krishna Pinisetty, Praveen Kumar Balguri, Anil Kumar Bandela, Udayabhaskararao Thumu

**Affiliations:** 1 Institute of Fundamental and Frontier Sciences, University of Electronic Science and Technology of China, Chengdu 610054, China; 2 Department of Aeronautical Engineering, Institute of Aeronautical Engineering, Hyderabad 500043, India; 3 Department of Chemistry, Ben Gurion University of the Negev, Beer Sheva 84105, Israel

**Keywords:** cesium lead iodides, solar cell, perovskite stability, surface engineering, perovskite crystal structures, CsPbI_3_ NCs, ion exchanges, photoluminescence, defect-tolerance, hot-injection method

## Abstract

Among perovskites, the research on cesium lead iodides (CsPbI_3_) has attracted a large research community, owing to their all-inorganic nature and promising solar cell performance. Typically, the CsPbI_3_ solar cell devices are prepared at various heterojunctions, and working at fluctuating temperatures raises questions on the material stability-related performance of such devices. The fundamental studies reveal that their poor stability is due to a lower side deviation from Goldschmidt’s tolerance factor, causing weak chemical interactions within the crystal lattice. In the case of organic–inorganic hybrid perovskites, where their stability is related to the inherent chemical nature of the organic cations, which cannot be manipulated to improve the stability drastically whereas the stability of CsPbI_3_ is related to surface and lattice engineering. Thus, the challenges posed by CsPbI_3_ could be overcome by engineering the surface and inside the CsPbI_3_ crystal lattice. A few solutions have been proposed, including controlled crystal sizes, surface modifications, and lattice engineering. Various research groups have been working on these aspects and had accumulated a rich understanding of these materials. In this review, at first, we survey the fundamental aspects of CsPbI_3_ polymorphs structure, highlighting the superiority of CsPbI_3_ over other halide systems, stability, the factors (temperature, polarity, and size influence) leading to their phase transformations, and electronic band structure along with the important property of the defect tolerance nature. Fortunately, the factors stabilizing the most effective phases are achieved through a size reduction and the efficient surface passivation on the delicate CsPbI_3_ nanocrystal surfaces. In the following section, we have provided the up-to-date surface passivating methods to suppress the non-radiative process for near-unity photoluminescence quantum yield, while maintaining their optically active phases, especially through molecular links (ligands, polymers, zwitterions, polymers) and inorganic halides. We have also provided recent advances to the efficient synthetic protocols for optically active CsPbI_3_ NC phases to use readily for solar cell applications. The nanocrystal purification techniques are challenging and had a significant effect on the device performances. In part, we summarized the CsPbI_3_-related solar cell device performances with respect to the device fabrication methods. At the end, we provide a brief outlook on the view of surface and lattice engineering in CsPbI_3_ NCs for advancing the enhanced stability which is crucial for superior optical and light applications.

## 1. Introduction

With the increasing demand for mankind’s energy needs, it is essential to develop sustainable energy technologies, such as the utilization of natural resources, for example, solar power. There has been an immense search, over several decades, for potential materials for highly efficient optoelectronic devices. These efforts were successful in a few forms of materials; among such potential candidates is lead iodide perovskites (ABX_3_; A = methylammonium cation, MA, formamide cation (FA) or Cs^+^; B = Pb^2+^; and X = I, that can be replaced by Cl^−^, Br^−^, or their mixtures). Importantly, the perovskites have emerged as superior materials due to their abundance, easier processing methods, and low-cost solutions for optoelectronic applications. Based on the chemical nature (organic (MA or FA) or inorganic (Cs^+^)), there is an opportunity for various A-site monocations in the unit cell of ABX_3_, and they are classified as “hybrid organic–inorganic” or “all-inorganic” perovskites. The initial all-inorganic ABX_3_ synthesis goes back to the 18th century, when it was demonstrated by Wells [1]. Their detailed crystal structure was further studied by Weber in the 19th century [2,3]. The field of perovskites attracted explicit attention at the beginning of the 20th century, because of the first perovskite-based solar cell (PSC) fabrication, which further took nearly a decade more to attract a broad scientific community [4]. The first solar cell application of perovskites (MAPbI_3_) was published by Kojima et al. in 2009 [5], and later on, this research accelerated exponentially with a substantial increase in scientific publications over the years, especially since 2015. The reason behind the greater attention toward ABI_3_ perovskites is due to their ideal band gap properties for light-harvesting applications [6,7]. In addition, intriguing properties, such as the long electron-hole diffusion lengths, high photoluminescence quantum yields (PLQY), and high absorption coefficients, have created tremendous interest in optical devices, furthermore, to be studied in its fundamental aspects in the interdisciplinary field of the material research communities [8].

**Figure 1:**
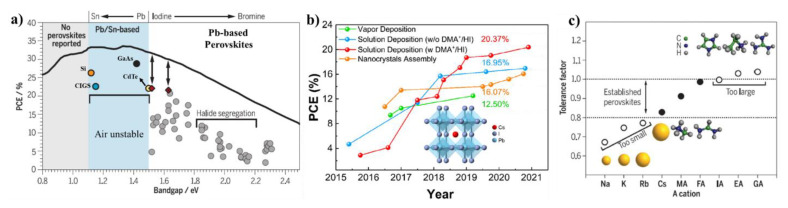
(**a**) Comparison of PCE among different materials, such as Si, CIGS, CdTe, GaAs, and perovskites. Note that APbI_3_ PSCs show higher PCEs than the APbBr_3_; (**b**) PCE performance of CsPbI_3_ PSCs fabricated using various processing methods; (**c**) Tolerance factor of APbI_3_ perovskites for various sized A cations. (**a**,**c**) adapted with permission from Ref. [17], Copyright 2017 from American Association for the Advancement of Science (AAAS) (**b**), adopted with permission from Ref. [10], Copyright 2021 Elsevier.

For the photovoltaic studies, the Shockley–Queisser limit is an important factor that is calculated by analyzing the quantity of electricity produced from the known amount of solar energy. The estimated maximum possible power conversion efficiency (PCE) is not more than 33.7% for a single p–n junction at an ideal band gap of 1.37 eV (using an AM 1.5 solar spectrum) [9]. For information, the band gap of the silicon in the solar cells is around 1.1 eV, and its highest theoretical PCE is 30%. In the lead halide perovskites, the bandgaps are tunable over a wide visible wavelength range (300 to 800 nm), depending on the type of halide composition (Cl, Br, and I or their mixtures). It is worth noting that all of the perovskite materials are not equally important for solar cell applications. Figure 1a shows that the materials with an electronic band gap between 1.1 to 1.4 eV are efficient to utilize the solar spectrum to generate electric power. From this Shockley–Queisser estimation, as well as the experimental performance, the low band gap perovskites in particular, APbI_3_ (1.73 eV), are regarded as one of the most efficient light-absorbing layers than the other wide-band-gap all-inorganic perovskites (APbBr_3_ (2.36 eV) and APbCl_3_ (3.0 eV)) [10,11,12]. The best performance for the thin film-based CsPbBr_3_ PSCs are around 10% [13], whereas the CsPbI_3_ performs at above 20% [14]. Due to its strong light absorption capability, a much-reduced thickness is sufficient for the utilization of photons to exhibit a better PCE [15,16].

A general question arises as to what would be the choice of ions (A, B, and X) to achieve efficient PCE in a given perovskite. In this context, the requirement of a suitable narrow bandgap is largely contributed by “iodide” in APbI_3_. Figure 1a shows the mixed halide (Br^−^ and I^−^) perovskites also struggle to reach the efficient PCE values of the pure iodide-based perovskites. The lower performance in the mixed halide perovskites is due to the halide segregation-related issues, which contribute to the nonradiative recombination process. Thus, in search of potential candidacy, the selection of halide X goes to “I” for many of the efficient solar cells. Next, for element B; for this selection, the Pb^2+^ ions exhibit a reduced nonradiative recombination which facilitates an excellent PLQY. This motivates the use of B to Pb^2+^. When it comes to the choice of cations, A, MA, FA, and Cs^+^ are excellent monovalent cations to create a three-dimensional (3D) network in a perovskite with excellent transport properties [18]. FAPbI_3_ is an excellent candidate, whose bandgap is at 1.48 eV. This FAPbI_3_ performed excellent solar cell efficiency at 20% [18]. The solar cell made from the MAPbI_3_ (1.1 eV) is an ideal candidate for the absorption layer, and also provides a PCE of 25%. Similarly, the CsPbI_3_ (1.73 eV) also acts as a suitable candidate for the solar cell devices. Among the individual MA, FA, and Cs^+^ perovskites, the MA-based perovskites show better optoelectronic properties. Still, in recent years, the Cs, FA-based perovskites are considered superior to MAPbI_3_, due to their improved stability [19,20,21]. The attraction of the CsPbI_3_ originates mostly from their complete inorganic nature [22,23,24]. The stability comparisons among these systems (MAPbI_3_, FAPbI_3_, and CsPbI_3_) are described in detail in the later sections.

From the above discussion, it is clear that the all-inorganic CsPbI_3_ is thermally stable than the iodide-based hybrid perovskites. However, the optically active α-black CsPbI_3_ undergoes undesired lattice transformations towards the δ-yellow CsPbI_3_ phase (non-perovskite structure) upon prolonged storage at room temperature (RT). This structural transformation is unavoidable, due to the high flexibility in the perovskite lattice and the low formation energy of the δ-phase at RT [25,26,27]. Subsequently, to solve these issues, many researchers investigated their crystal properties, developed protocols to stabilize for optically active CsPbI_3_ NCs, explored their phase stability (under the influence of thermal and moisture), post-synthetic surface treatment methods, the doping of foreign ions, strain control through size effect, and surface engineering, which offered tremendous interest for providing further improvements in the photovoltaic efficiencies.

This review specifically covers the research aspects of CsPbI_3_ and provides up-to-date developments in the field of CsPbI_3_, including crystal structural properties, band structure, size-related stability. The comprehensive understanding of the CsPbI_3_ system can be obtained from the scientific knowledge gained from bulk as well as their nanocrystal forms. For example, X-ray diffraction (XRD) and the advanced diffraction techniques are highly feasible on bulk CsPbI_3_, whereas high resolution atomic structure analysis is feasible at their nanoscale. So, throughout the text, we use the CsPbI_3_ and CsPbI_3_ NCs to represent the bulk and nanoforms of their systems, respectively. Additionally, this review also provides the recent efforts in enhancing the stability of CsPbI_3_ through size reduction and improved optical properties toward humidity, heat, and harsh environments, through the suppression of the surface defects and the maintenance of the ideal tolerance factor through pre- and post-synthetic surface passivation by surface engineering via the molecular structures and inorganic salts. Finally, we provided the future directions and opportunities for the CsPbI_3_ system.

## 2. Brief History of CsPbI_3_ to a Powerful Solar Cell Device

The history of the CsPbX_3_ materials, even their zero-dimensional variant Cs_4_PbX_6_, began two decades earlier than the name perovskite was given. These materials were first introduced by Wells in 1893 [1], and Moller (1957–1959) [28]. The cesium lead iodide (CsPbI_3_) was discovered by Weber in 1978 [3]. The earlier research was focused on studying the optical properties during doping stoichiometric amounts of Pb^2+^ in the CsX matrix [29]. The interplay between a stoichiometric mixture of the reactants and the temperature resulted in the formation of CsPbI_3_, later added to the perovskite family. After a gap of several decades, for the first time the hybrid inorganic–organic halide perovskites (MAPbI_3_) were introduced and used as a sensitizer in a liquid electrolyte (2009). Surprisingly, the cell performed at a PCE of 4.5% even in their initial efforts [5]. This contribution made its mark in the material and energy research field around the world in the last decade until the present date. The first perovskite solid-state solar cell that worked in an open-air environment was reported by Kim et al. in 2012, with a notable stability of 500 h, and exhibited an impressive PCE of 9.7% [30]. The NREL-certified PCE of such solar cells is above 25%, making them the most impactful products in the field of solar energy [31]. Beyond these applications, the strong PLQY in APbI_3_ NCs with tunable emission wavelengths and high color purities, proved their advantages in light-emitting diode and laser applications.

The first CsPbI_3_ PSC with a PCE of 2.9% was fabricated in 2015 by Snaith and coworkers [32]. After the successful introduction of CsPbI_3_ as an absorbing layer, numerous methods were developed with novel processing methods to fabricate high-quality CsPbI_3_ PSCs resulting in rapid progress in the field of CsPbI_3_ SCs. Based on the processing method and the performance during the evolution of CsPbI_3_ PSCs, a few breakthroughs are listed below (Figure 1b): (i) chemical vapor deposition method to achieve PCE up to 12.50% [33]; (ii) Deposition of CsPbI_3_ NCs with at PCE up to 16.07% [34]; (iii) solution deposition with and without dimethylammonium (DMA^+^)/hydroiodic acid (HI) additive exhibiting PCE up to 20.37% and 16.95%, respectively [14,35]. The best-performed PCE value of the CsPbI_3_ SCs reported so far is around ~20% [36]. This PCE is relatively on the lower side but comparable to those of the MAPbI_3_ PSCs (up to 25%). The primary reason for the relatively lower favorable photophysical properties of the CsPbI_3_ is because of its relatively larger bandgap (1.74 eV) compared to the MA- (1.3 eV), or the FA (1.4 eV)-based perovskites [37]. Note that the relative increase in the band gap leads far from the ideal Shockley–Queisser estimated band gap (1.37 eV). The higher band gap of the CsPbI_3_ (1.74 eV) compared to the hybrid perovskites results in relatively lower PCE values. However, the substantial advantages arise from considering their stability compared to the hybrid perovskites [37]. Additionally, the CsPbI_3_ with a high electron diffusion carrier, the defect tolerance, the large absorption cross-sections, and the large-scale solution processability at a low cost offers the production of high PCE of solar cells at an industrial level.

As mentioned in the introductory section, the α-CsPbI_3_ (the thin films as well as NCs) is emerging as an excellent light-absorbing material. The α-CsPbI_3_, even at their high defect states (~10^16^ cm^−3^), show an excellent performance over the conventional PbS QDs, in view of their conductive and defect-tolerant electronic structure (discussed in Section 8) and longer carrier lifetime (~2 ns). In addition, the α-CsPbI_3_ NCs also shows higher hole mobility (μ~0.2–0.5 cm^2^/V·s) than that of the PbS QDs [38,39]. Another challenge faced by these PbS QDs is the production of multiple-charge carriers at beyond threshold energy (typically ~3 Eg), and at such energies the performance of the charge transport layer is poor [40]. Interestingly, the high-yield production of multiple-charge carriers without the requirement of threshold energy which results the charge transport layer further supports the improved PCEs. Due to these advantages, for example, the PCEs of the α-CsPbI_3_ NC solar cell has recently been demonstrated to reach a record value, as listed in Table 1. Various deposition methods, such as vapor deposition, solution deposition, layer by layer deposition, and spray deposition methods have been widely used for the fabrication of thin film-based CsPbI_3_ SC whereas the NC self-assembly followed by the shorter ligand post-modification methods (discussed in the following sections) were employed for the CsPbI_3_ NC SC. Even though the bulk α-CsPbI_3_ thin films have exhibited higher PCE values, it is not easy to maintain the desired cubic structure in the bulk film and the stability is improved only at smaller sizes.

## 3. Structural Stability of CsPbI_3_ Perovskites

The crystal structure has a remarkable role in determining the photo-response characteristics of CsPbI_3_. The cubic structure (α phase/black phase) of CsPbI_3_ is considered as an ideal 3D material (inset of Figure 1b). The smaller Pb^2+^ cation is stabilized in the octahedral site shared with six I anions [PbI_6_]^4−^, while the larger Cs^+^ occupies the cubic octahedral void (middle of the eight shared [PbI_6_]^4−^ octahedra) coordinated with twelve I anions. The stability and deviation of a perfect perovskite crystal are empirically predicted by the dimensionless descriptors, Goldschmidt’s factor (*t*) and octahedral factor (*μ*) [41,42,43,44]. The three structural polymorphs of CsPbI_3_ are identified, and all of these ‘black’ phases exhibit excellent photovoltaic properties. These photoactive “black” CsPbI_3_ perovskite phases are α-phase (cubic Pm3m); β-phase (tetragonal P4/mbm); γ-phase (orthorhombic Pbnm), and one non-photoactive δ-phase (orthorhombic, Pnma).

Octahedral factor:

*μ* = *r*_B_/*r*_X_

Goldschmidt’s tolerance:

$$t=\frac{r_{A}+r_{X}}{\surd 2\left(r_{B}+r_{X}\right)}$$

where *r*_A_, *r*_B_, and *r*_X_ are the ionic radii of the A, B, and X-site ions of the ABX_3_ structure, respectively. “*t*” is also defined as the ratio of the distance A−X to the distance B−X. These two factors evaluate how efficiently the crystal lattice is filled in the structure. For an ideal ABX_3_, the value of *μ* lying in the range of 0.44 < *μ* < 0.90 tends to stabilize [BX_6_]^4−^ octahedra [45]. As shown in Figure 1c, a stable 3D perovskite phase can form when the value of “*t*” lies within the range 0.8 < *t* < 1.0. If it is computed outside this range (*t* > 1.0 or *t* < 0.8), the perovskite is not capable of maintaining its 3D structure and forms a low-dimensional material, where the [PbI_6_]^4−^ octahedra are arranged in layered 2D perovskites, 1D chains, or 0D clusters at RT. The greater tolerance factor (*t* > 1.0), implies that the size of the A-site ion is excessively large compared to the octahedral site created by [PbI_6_]^4−^ corner sharing. The excessive strain in the lattice, disassembles the 3D octahedral mesh into various kinds of layered-2D perovskites, such as L_2_PbI_4_ (L= large sized cation). If the tolerance factor is close to 0.8 (*t* = 0.8), it initially undergoes transitions to tetragonal or orthorhombic phases through [PbI_6_]^4−^ octahedra rotations, and finally leads to thermodynamically stable non-perovskite phases at RT. When “*t*” is too small (*t* < 0.8), the smaller A-site ions filled in the octahedral gap cannot effectively support the octahedral network. A calculated tolerance factor of 0.8 for CsPI_3_, in this case, the larger size of the octahedral void in comparison to the Cs^+^ ions (1.88 Å) leads to the rattling of the Cs^+^ ions in the soft ionic crystal lattice of α-CsPbI_3_, and perturbance of the Coulombic interactions causes lattice distortion.

**Figure 2:**
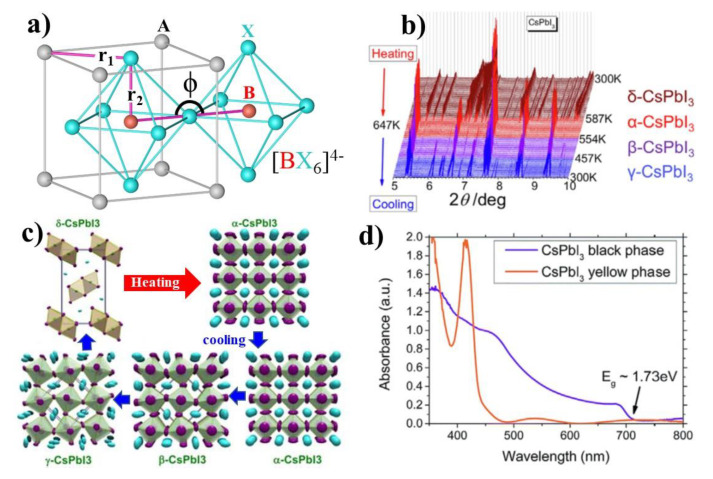
(**a**) Crystal structure of ABX_3_. The label r_1_ = r_A−x_, r_2_ = r_B−x_ and ϕ = B−X−B (Pb−I−Pb); (**b**) Synchrotron XRD patterns of CsPbI_3_ at different temperatures. (**c**) Schematic illustration of crystal phase transitions in CsPbI_3_ upon temperature cycle. (**b**,**c**), adopted with permission from Ref. [53], Copyright 2018 American Chemical Society. (**d**) UV–vis absorption spectra of black-phase and yellow-phase CsPbI_3_ thin films. Adopted with permission from Ref. [32], Copyright 2015 Royal Society of Chemistry.

This vibration in the octahedral mesh results in the tilt of the [PbI_6_]^4−^ which can lead to the transformation from corner-sharing α-CsPbI_3_ to edge-sharing δ-CsPbI_3_ (non-perovskite, strain-free orthorhombic yellow phase). The changes in the crystal lattice cause a dynamic shift in the band edges (Figure 2d), therefore the narrow band gap in δ-CsPbI_3_ (1.74 eV) changes to a wide bandgap of 2.82 eV, which now acts as an inactive layer for photon absorption, due to their poor optical properties [46,47,48,49]. This undesired structural transformation process is accelerated in the presence of moisture at RT. It is worth mentioning that these “*t*” and “*μ*” estimations could only provide a basic understanding but are not necessary to predict the in-depth energetically favored crystal phases. It is because these “*t*” and “*μ*” are estimated from a geometrical point of view rather than considering all of the chemical and dynamic interactions to define the lattice stability of a perovskite [50,51,52].

## 4. Comparison of Hybrid and All-Inorganic Perovskite Stability

The research on perovskites takes the advantages of XRD analysis to investigate kinetic changes, time-dependent reaction processes, and elucidate the degradation mechanisms. The XRD analysis provides the thermal stability of the three major APbI_3_ systems; the obtained thermal stability is in the following order MAPbX_3_ << FAPbX_3_ < CsPbX_3_. This stability is associated with the volatility of the A-site cation halides decreasing in the order of MAX << FAX < CsX. It implies that MAX is more volatile than CsX explains the reason for the higher decomposition rates for MAPbI_3_ than the CsPbI_3_. In a typical photovoltaic device, the operating temperature usually surpasses 333 K; thus, preserving the long-term stability at these temperatures is crucial for industrial applications. It is well known that, even without applying external heat stress, the MAPbI_3_ was already intrinsically unstable. At the moisture conditions, the MAPbI_3_ chemically decomposes to MAI and PbI_2_ at RT [54]. The decomposed structures δ phases vary with the type of cation, for example, hexagonal for δ-FAPbI_3_ and orthorhombic for δ-CsPbI_3_ [55].

Next, the discussion focuses on a few advantages of the Cs^+^ cation over the organic cations. By replacing MA with Cs^+^, the formation enthalpy of CsPbI_3_ at 0 K decomposition was increased to ~0.1 eV, which indicated that the CsPbI_3_ is intrinsically stable [25]. The absence of proton in the alkali metal Cs^+^ prohibits the deprotonation reactions (e.g., free electrons and hot carriers) that commonly occur in the perovskites. Another disadvantage for MA and FA is their inherent hygroscopic nature, which causes high reactivity toward moisture. Due to this inherent nature, the hybrid perovskites undergo hydrolysis in moisture conditions, which release the Coulombic interaction between the cation and the inorganic octahedra. The FA cation chemically dissociates into s-triazine (HCN)_3_ and ammonia in the presence of acidic water, while Cs^+^, being inorganic, can survive in such chemical impacts. Another factor influencing the crystal structure is the orientation freedom of the A-site cation. The asymmetric organic cations (CH_3_-NH_3_^+^) rotate within the octahedral void, which makes the hybrid perovskites further unstable. This rotation also affects the nature of the band gap, while the Cs^+^ is symmetric without multiple-band structures. However, there are also a few lower sides for the effectiveness of the CsPbI_3_. The major factor lies in its relatively low tolerance factor (*t* = 0.8). The Cs^+^ ionic radius, (Cs^+^ = 1.88 Å), is smaller compared to that of the most popular organic cations, including MA (2.53 Å) and FA (2.17 Å). The energy to retain the cubic structure for the larger A-cations (MA and FA with *t* > 1) is lower compared to that of the smaller Cs^+^ ions. As a result, the optically active α-CsPbI_3_ is only stable at very high temperatures (T = 300–360 °C) [32], higher than that of α-FAPbI_3_ (T = 150−185 °C), and α-MAPbI_3_ (T = 100 °C) [56,57]. The variation in these cations had a profound effect on the formation of other polymorph structures, for example, FAPbI_3_ the β- and γ- phases (−122 and −182 °C, respectively) were formed at substantially lower temperatures. Based on the above discussion, from a geometrical point of view, there is no single cation that cannot fully support the purpose of utilizing the long-term stable photovoltaic devices. Many of the researchers developed mixed cation approaches, such as Cs_x_FA_1−x_PbX_3_ to improve their stability, following their potential usage for light harvesting. As we discussed above, benefiting from the alkali metal nature of Cs^+^, and the complete inorganic nature of CsPbI_3_ is more intrinsically stable against light and heat stresses, which makes it more promising for practical application under working conditions.

## 5. Influence of Temperature on Crystal Phase of CsPbI_3_

The following discussion contains the real-time temperature-dependent crystallographic properties of CsPbI_3_, as reported by Marronnier et al. [53]. In this study, the δ-CsPbI_3_ starting from RT was heated above >310 °C for the transformation of α-CsPbI_3_. Figure 2b,c depicts the dynamic structural transformation of CsPbI_3_ during the heating cycle. During this transformation process, the unit cell volume got expanded as a result of the dynamic tilt in the [PbI_6_]^4−^ octahedra from an edge-sharing to a corner-sharing configuration [58,59]. After the complete transformation to α-CsPbI_3_, the temperature is gradually decreased, while analyzing the structure changes through Synchrotron XRD. The XRD results (Figure 2b) show that the α-phase does not return directly to its original δ-CsPbI_3_ phase, but progressed through the β- and γ-CsPbI_3_. During the cooling step, the α-phase first relaxes to tetragonal CsPbI_3_ (β phase, below 260 °C), followed by an orthorhombic CsPbI_3_ (γ-phase, at 175 °C). Finally, the γ-phase spontaneously relaxes to the thermodynamically stable δ-phase on the process of reaching RT [60]. The transformation kinetics highly depend on the temperature and humidity of the surroundings [53].

These three α-, β-, and γ-CsPbI_3_ phases possess a corner-sharing octahedra network, but differ in their Pb−I−Pb bond angles (ϕ, Figure 2a) and vary in their degree of symmetry [11]. Those corresponding structure models, the distortion of the Pb–I–Pb bond angles, and the diverse lattice parameters are schematically presented in Figure 2c. As shown in Figure 2a,c, the cubic structure of α-CsPbI_3_ displays the highest symmetry with a Pb−I−Pb bond angle of a perfect 180° (ϕ = 180°) i.e., the undistorted lead iodide octahedra [61]. These temperature-dependent results reveal that the highly crystal symmetric structures tend to be stable at higher temperatures. As the temperature decreases, the energy received to align the perfect bond angle decreases, in this context, the high-symmetry lattice of α-CsPbI_3_ is distorted by a reduction in the Pb−I−Pb tilting angle (ϕ = 164°; T ≈ 260 °C) results in the formation of β-CsPbI_3_. The reduced angle causes a tetragonal distortion of the corner-sharing lead iodide [PbI_6_]^4−^ octahedra. A further distortion of the β-CsPbI_3_ structure leads to a further lowering of the Pb−I−Pb bond angle, resulting in the formation of metastable γ-CsPbI_3_ (ϕ = 153°; T ≈ 175 °C) [62,63,64]. This finally transforms to δ-CsPbI_3_; it has a structure with the smallest Pb−I−Pb bond angles (95.09° and 91.40°) and comprises 1D double chains of [PbI_6_]^4−^ edge-sharing octahedra [65]. The variation in the Pb−I−Pb bond angles in each CsPbI_3_ polymorph changes the optoelectronic properties, such as the electronic band structure, the density of the states, exciton-binding energy, and the amount of polarity or ferroelectricity in the material, which in turn affect the device performance. The theoretical calculations reveal that the stability of the black phases is in this order γ-CsPbI_3_ > β-CsPbI_3_ > α-CsPbI_3_ at RT [66]. Besides, the reduction in the bond angle leads to a slight increase in the band gaps than the cubic α-CsPbI_3_ with maintaining the same property of direct band gap (discussed in Section 8) [35,65].

## 6. Polar Medium-Induced Transformation of α-CsPbI_3_ NCs to δ-CsPbI_3_

The structural studies at the atomic level are quite complex, due to the dynamic changes in the crystal structure under ambient conditions. The NCs provide better chances for atomically precise characterization, though there are some limitations, such as Scherrer broadening occurring due to small crystallite sizes. The CsPbI_3_ NCs undergo modification or degrade into metallic Pb nanoparticles under electron beam illumination. These limitations also challenge the atomically resolved characterization of the perovskite structure in a conventional TEM. Wan et al. studied the lattice transformation of the CsPbI_3_ NCs in the presence of a polar medium, through a careful investigation of the aberration-corrected STEM technique. For example, the cubic α-CsPbI_3_ and δ-CsPbI_3_ perovskites can be distinguished based on the arrangement of bright Pb-I atomic columns in their (100) lattice plane. These Pb-I atomic columns arrange in a perfect cubic in the case of the α-CsPbI_3_ NCs and a zig-zag fashion in the case of the orthorhombic δ-CsPbI_3_ NCs [67].

**Figure 3:**
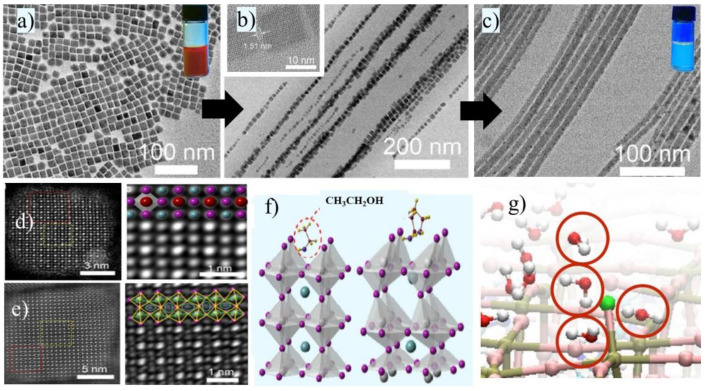
(**a**–**c**) TEM images showing the transformation of α-CsPbI_3_ nanocubes to δ-CsPbI_3_ nanowires during the reaction of α-CsPbI_3_ nanocubes with ethanol, insets show their respective photographs of nanocrystal solutions collected under UV light; (**d**,**e**) STEM images of α-CsPbI_3_ NCs and δ-CsPbI_3_ NCs; (**f**) Simulated model of ethanol molecules adsorbed on α-CsPbI_3_ NCs structure causing the structural distortion. The images (**a**–**f**) adapted with permission from Ref. [67]; Copyright 2018 American Chemical Society. (**g**) Simulated model shows a view of three H_2_O molecules lifting an iodide atom (depicted in green for clarity). Adapted with permission from Ref. [68]. Copyright 2017 American Chemical Society.

Figure 3a–f shows a mechanistic understanding of the polar medium-induced transformation of the α-CsPbI_3_ nanocubes to δ-CsPbI_3_ nanowires. This entire process was studied by sophisticated STEM and DFT analysis. Initially, the perfect distortion-free arrangement of Pb-I and Cs atomic columns are seen in α-CsPbI_3_ NCs (Figure 3d) which undergoes a structural transformation toward the zig-zag structure (Figure 3e), in the presence of ethanol or any other polar solvent. The authors claim that the ethanol adsorbs on the Cs^+^ ion, inducing distortion into the octahedra mesh. This distortion from the perfect arrangement of the [PbI_6_]^−^ octahedra leads to a reduction in the NC symmetry. In addition, the electron cloud on the [PbI_6_]^−^ polarizes to create a dipole moment in the system. Their permanent dipole moment in the asymmetric CsPbI_3_ nanocubes triggers them to attract the other nanotube with the opposite charges. This simultaneous transformation of the orthorhombic structure followed by the dipole-induced self-assembly results in the formation of the nanowire-shaped δ-CsPbI_3_. The rate of the transformation is proportional to the polarity of the solvent. However, an in-depth mechanistic understanding of this transformation is still not clear and yet to be explored. Caddeo et al. studied the influence of water vapor and liquid water on the MAPbI_3_ decomposition process through atomic simulations [68]. In the case of water vapor, the water molecule penetrates into the crystal lattice but there is no destruction to the cubic structure. This process of the shuttling of water molecules among the lattice and surroundings is reversible upon thermal evaporation. In the case of liquid water, a similar process occurs, where the H_2_O enters into the crystal lattice without altering the lattice parameters. However, the situation is different when the surface coverage of the H_2_O molecules is above 75%, the lattice starts to react with the water. As shown in Figure 3g, the decomposition process begins with three water molecules lifting the iodine followed by solvating the MA and I ions layer by layer, leading to the formation of insoluble PbI_2_ films (MAPbI_3_ finally to decomposed to MAI and PbI_2_). Hence, the complete collapse of the cubic structure is a result of the collective water molecule effect.

## 7. Controlling Surface Energy to Stabilize the Black Phase CsPbI_3_

**Figure 4:**
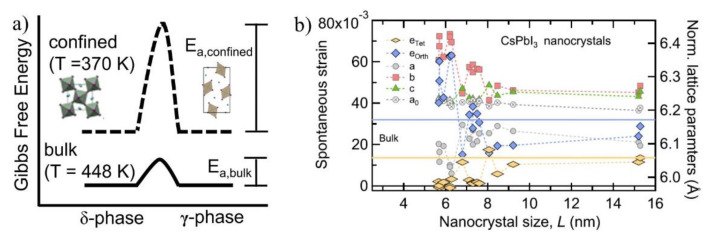
(**a**) Energy diagram representation to differentiate the α-to-δ phase transition in bulk CsPbI_3_ and CsPbI_3_ NCs (confined). Adapted with permission from Ref. [73]; Copyright 2019 American Chemical Society. (**b**) Normalized lattice parameters and corresponding spontaneous strains found in CsPbI_3_ NCs for various crystal sizes (L). Adapted with permission from Ref. [41]. Copyright 2021 American Chemical Society.

The recent studies prove that the stability of the black phase CsPbI_3_ at their nanoscale is far better than that of the bulk materials (Figure 4a). One such example is that the CsPbI_3_ NCs exhibited a notable stability against the lattice degradation, compared to the bulk upon continuous illumination [69]. Even in their bulk phase, this undesired transformation is highly influenced by the grain size [70]. This additional stability of the CsPbI_3_ NCs is due to their higher surface tension, as a result of the large surface-to-volume ratio at their nanoscale, which alters the formation energies of the crystal phases [71]. Zhao et al. reported, in the case of CsPbI_3_ NCs, a reduction in the lattice parameters and an increase in the tensile surface strain from the size changes from 15 nm to 3 nm [41,72].

The surface energies exhibited 3–5.1 eV for the CsPbI_3_ NCs of a size of 15.3–5.2 nm, whereas this value was negligible for the large NCs or bulk films. In other words, the difference in the surface energy is stored in the form of the high tensile strain of the NCs. This increased tensile strain supports the octahedral tilting as small as possible to obtain a high symmetric black phase at RT [72,74]. In bulk, when the surface energy is not accountable, the formation energies of the phases are in this order *α* > *γ* > *δ*. Interestingly, as the sizes become smaller and smaller, the rise in the surface energy reverses the thermodynamic stability of these phases *α* > *γ* > *δ*. For example, at below 5.6 nm, the *γ*-phase NC is more thermally stable than the yellow *δ*-phase, further down to 2.6 nm stabilizes the *α*-phase more than the other phases. Among the perovskite phases (α, β, and γ), the CsPbI_3_ NCs around 10 nm possess the γ phase, rather than α. Consequently, an analysis of the lattice constants and the structural strain as a function of size is presented in Figure 4b. It is very clear that the strain components are constant from the 16 nm- to the 10 nm-sized CsPbI_3_ NCs. When the sizes reach below 10 nm, a dramatic change is seen in their spontaneous strain parameters, indicating the breakdown of the bulk-like structural behavior. Remarkably, at 5 nm, the NCs eliminated the ϵ_tet_ strain and shifted to another direction (ϵ_orth_), and its value is doubled near 5 nm. The structural properties sized below 10 nm could be well understood by the spontaneous strain model i.e., at this size scale, the surface energy started to contribute to the Gibbs free energy to control the overall phase stability [41]. Due to the advantages of the CsPbI_3_ NCs at smaller sizes for stable performance, there are various synthetic strategies to regulate the perovskite grain size for device fabrication: (i) utilizing pristine CsPbI_3_ NCs directly to deposit on substrates [75,76]; (ii) in-situ regulation of the grain size of perovskite through additives during its growth [36,77,78,79]. Due to the improved stability at their nanoscale, the initial control of the black phase-CsPbI_3_ could be dictated through well-designed synthetic protocols, however, the undesired yellow phase transformation is occurring over time. The current challenge is to stabilize the preformed CsPbI_3_ NCs in their original optical active forms. The current strategy to retain the existing structure is to concentrate the lattice, which favors the interactions between the octahedral void and Cs^+^ ion. In addition to the above factor, the CsPbI_3_ NCs passivated by the organic ligands increase the colloidal stability by resisting their coalescence and degradation [72]. The higher stability of the CsPbI_3_ NCs protected by the cationic/anionic ligands is likely also explained based on the increased tolerance factor, by considering the ligand-cations into its equation, as the ligands cover half of the surface.

## 8. Electronic Band Structure and Defect Tolerance

**Figure 5:**
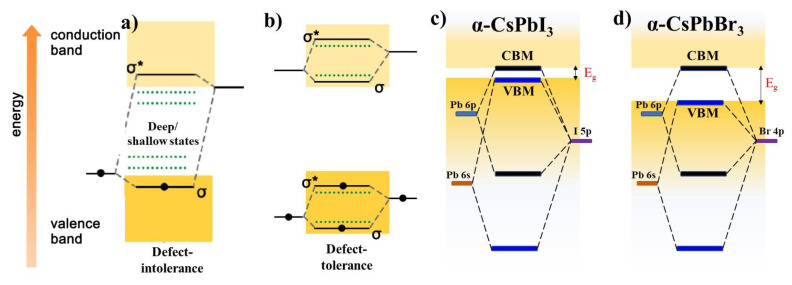
A model electronic band structure of defect-intolerance (**a**) and defect-tolerance (**b**) band structures. σ and σ*, represent the bonding and antibonding orbitals, respectively. (**b**) Schematic of the band structure of α-CsPbI_3_ showing the tendency to form traps or shallow states within the conduction band minimum (CBM) and valence band maximum (VBM). (**c**,**d**) electronic band structure of CsPbI_3_ and CsPbBr_3_ (**a**–**d**) are redrawn from Ref. [88], Copyright 2017 American Chemical Society.

CsPbI_3_ belongs to a family of direct band-gap semiconductor materials with efficient electron-hole pairs generation upon light absorption [22,32]. It has a band gap of 1.74 eV as a result of the electronic transactions between an empty Pb-p orbital (CBM) and a fully-occupied I-p orbital (VBM). The band gap of the upper VB and lower CB is formed by the antibonding orbitals. The outer electronic configuration of Pb(II) is 6s^2^6p^0^, and it is 5p^6^ for iodide. In brief, the CB (Pb 6p) mainly consists of the antibonding orbitals of Pb 6p and a negligible contribution from the iodide 5p orbitals, hence is predominantly a Pb p character [80]. The upper valence band (Pb 6s-I 5p) is made of fully occupied antibonding Pb 6s^2^ and a major contribution from the I-5p orbitals, conferring on the band a partial s-type character. These octahedra interactions infer that, while light harvesting, the I-5p electrons can be photo-excited to Pb-6p empty states that are dipole allowed [81]. Overall, it opens a band gap between two antibonding orbitals as a result of its dominant ionic nature. The CsPbI_3_ exhibits a direct band gap located at the R symmetry point of the Brillouin zone. The unique bandgap between antibonding orbitals also results of spin-orbit coupling. In brief, the VBM and CBM are moved slightly from R as a result of the spin–orbit coupling, which is strong in the case of the heavy elements (Pb and I). This coupling allows the energy level to split strongly, resulting in the increase in the width of the band gap [82]. This splitting is highly beneficial to bring the lower conduction band below the Pb(6p) atomic orbital. In this way, the CsPbI_3_ takes advantage of where the defect states (halide vacancies) stay within the band (VBM and CBM) states (Figure 5a,b). In contrast, in the case of semiconductors (Si, CdSe or GaAs) the defect states stay middle of the VBM and CBM (Figure 5a). The DFT calculation has demonstrated that the perovskites show no in-gap defect states, but rather the defect states appear as resonances inside shallow transition levels within the bands. The usual deep and shallow states are the presence of low formation energy defects, such as vacancies, interstitial atoms, and surface states. Due to this unique band structure in CsPbI_3_, in contrast to the defect-intolerant nature in the traditional semiconductor quantum dots, the presence of the surface defect states had little influence on the radiative recombination process and exhibited an excellent PL performance [83,84,85]. It is worth noting that years of in-depth studies on the surface defects revealed that the main contribution to the non-radiative recombination originated from the halide vacancies (V_X_) rather than the lead vacancies (V_Pb_). There are also a few reports showing that these defects in NCs can be overcome by a self-purification mechanism [70,86,87].

This kind of defect tolerance property is due to the band structure created from the octahedra lattice, rather than the orbitals of the Cs^+^ cation. Therefore, the A-site cation (MA, FA, or Cs^+^) orbitals had no direct overlaps to support the electronic band structure, but they could indirectly alter the band gap by causing changes in the crystal structure as a result of the [PbI_6_]^4−^ octahedra tilting [89]. Since the VBM is predominantly provided by the presence of the halogen-d orbital, an obvious shift in the position occurs when changing the type of halide ions (Figure 5c,d). For example, introducing Br^−^ in CsPbI_3_ increases the band gap, which is mainly caused by a downshift of the upper valence band while there is no significant movement in the CBM [80]. Besides the electronic structure, the crystal phases (α, β, γ, and δ) also influence defect tolerance because of variation in their I-Pb-I bond angles. The overlapping between Pb and I orbitals is highly related to that of the tilt in the [PbI_6_]^4−^ octahedra. The smallest bond angle in the δ-phase weakens the antibonding character of VBM, which results from a poor defect tolerance property. Due to this reason, the δ phase exhibits deeper defect-transition energy levels than the γ phase [90].

## 9. Surface Defect Passivation Methods

**Figure 6:**
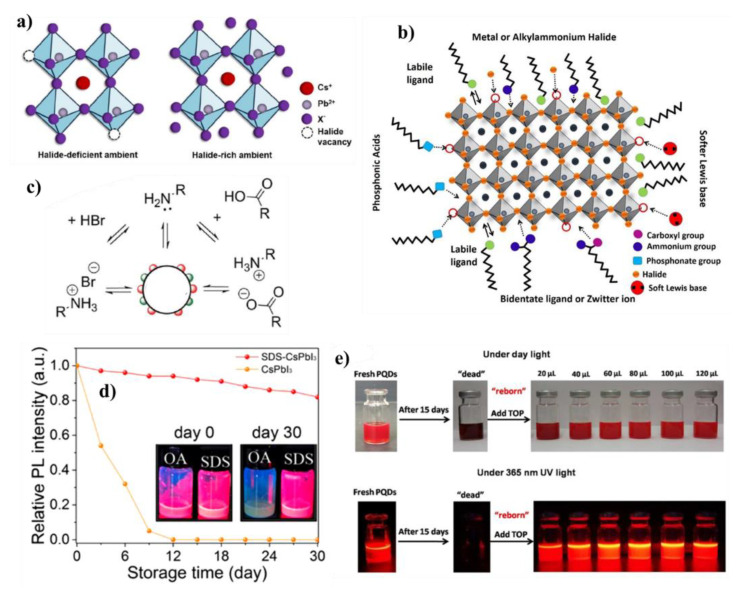
(**a**) Schematic representation of CsPbI_3_ NCs in halide-deficient and halide-rich perovskite crystal; (**b**) Schematic depiction of surface passivation by capping ligands with different functional groups. (**a**,**b**) are adapted with permission from Ref. [98], Copyright 2019 American Chemical Society. (**c**) Schematic representation of the dynamic surface stabilization by oleylammonium bromide, oleylammonium oleate, and oleylamine. In addition, the relevant acid/base equilibria are depicted. Adapted with permission from Ref. [96], Copyright 2016 American Chemical Society. (**d**) Normalized PL intensity of CsPbI_3_ NCs with time for both SDS-treated and untreated nanocrystals. Adapted with permission from Ref. [99], Copyright 2021 American Chemical Society. (**e**) Photographs of the quenched PL emission from CsPbI_3_ NCs regained after the addition of the TOP ligand. Adapted with permission from Ref. [100], Copyright 2018 American Chemical Society.

At the nanoscale, as compared to the bulk, the fraction of the surface atoms is predominately exposed, resulting in the generation of many surface defects (Vx, V_B_, and V_A_) as a result of the incomplete coordination of the surface atoms. A large excessive number of such defect energy levels dominates over the defect-tolerance property, resultsthe formation of excessive exciton traps which contributes to the non-radiative recombination for the low PLQYs. Among these defects, the Vx defects are far more damaging to their optical properties. These trap states could be successfully suppressed by suitable surface passivation, or creating core-shell structures as in the case of conventional semiconductor QDs [91]. Interestingly, the CsPbI_3_ NCs with a high QY (~100%) can be achieved easily by ligand passivation or by creating halide-rich surfaces [85,92]. This phenomenon is related to the high defect tolerance of these materials, as discussed above [83,88]. Figure 6a represents the common Vx defects and the halide-rich surfaces in perovskites. Here, two commonly used passivation approaches are discussed; passivation while synthesizing the NCs, and post-synthesis treatment.

### 9.1. Nature and Interaction of Ligands on NC Surface

The ligand anchoring groups play a crucial role in the CsPbI_3_ NC stability. Unlike in the case of noble metal and semiconductor NCs, the bonding nature of the ligand on CsPbI_3_ NCs is highly dynamic, i.e., it can leave and attach to the NC surface for sensitive changes [93]. For example, at higher dilutions, the binding ligands detach from that of the NC surface, consequently, the greater number of halide-defect surfaces (V_X_). Based on the NMR studies of ligand dynamics, the defect tolerance values of CsPbI_3_ NCs, CsPbBr_3_ NCs, and CsPbCl_3_ NCs, are 9500, 390, and 53, respectively [94]. To achieve long-term stability, it is necessary to minimize the dynamic nature of the ligand and maintain a good binding constant. The binding ligands are classified into three types, based on their type of bonding, X-type, L-type, and Z-type (Figure 6b).

The commonly used X-type ligands to synthesize colloidal CsPbI_3_ NCs are oleylamine (OLAm) and oleic acid (OA) [95]. De Roo et al. [96] performed ^1^H NMR spectroscopic studies on these NCs. The results show that the octadecene and OA cannot bind by themselves, while oleylammonium bromide was proposed as the main capping ligand. Three possible combinations of these ligands were then proposed: oleylammonium bromide, oleylammonium oleate; and the OLAm (L-type ligand). The increase in the number of amines in the solution shifts the acid–base equilibria, which improves the binding nature of the oleate on the NC surface (Figure 6c). In this way, the amine plays many crucial roles in controlling the acid-base equilibria. Another key role of the OLAm is to suppress the defects of Cs^+^ vacancies through the substitution of OLAm cation (ammonium ions) in the Cs^+^ defect surface, via the assistance of the hydrogen bonds [94]. It is important to note that the excess of OLAm could deform the NCs through surface reconstruction, leading to a blue shift in their emission. However, due to the dynamic binding nature, the structure of the surface ligands is still unclear, for example, there is amine-free method to prepare stable CsPbX_3_ NCs passivated by OA [97]. In another report, the authors successfully stabilized the CsPbI_3_ NCs with high PLQY (80 to 95%), using dicarboxylic acid in a post-synthetic treatment. This bifunctional ligand is simultaneously attached to two Pb ions of the NC surface with an increase in the binding energy of 3.5%, compared to OA [92].

The ligand design and their steric hindrance had a strong impact on the NC morphology, and the crystalline phase. The structure of the ligand also protects the surface from undesired reactants, such as O_2_ and moisture [101]. The ion vacancies, migrations, or un-coordinated ions cause a certain degree of acidity or basicity on the NC surface. In a CsPbI_3_ NC, both the Cs^+^, as well as the Pb^2+^, are of a weak acid, and I^−^ a weak base [102]. Thus, one needs to consider the suitable degree of acidity and basicity of the ligands for effective passivation. For instance, weak acid defects are suppressed by a weak base, while weak base defects neutralize a weak acid. This could be explained by FAPbI_3_ films, its cubic structure stabilized better by the passivation of a weak base, phenylalkylammonium cation, compare to other protonated amines (aniline, and benzylamine), due to the interaction between NH_3_^+^ and I^−^ leading to effective passivation. The basicity of these amines after protonation is in the order of phenethylammonium cation (pKa 9.83) < benzylammonium cation (pKa 9.34) < arylammonium ion, PhNH_3_^+^ (pKa 4.87) [103]. In another study, Zhang et al. [104] reported the CsPbI_3_ NCs passivated with the C_8_ pair of ligands (octanoic acid or octylamine), show a higher stability than those of the pure C_18_ ligand pairs. This is explained based on the higher concentrations of −COO^−^ and −NH_3_^+^ from C_8_ ligands for the effective passivation of Cs^+^, Pb^2+^, and X^−^ defects. The synergistic use of the long-chain and short-chain ligands promoted the dispersion of the α-CsPbI_3_ NCs in solution and not only produced strong PLQY along with excellent upconversion luminescence properties, but also showed dual-mode luminescent characteristics [105]. Various types of single-head ligands have been used as surface passivating ligands, such as alkylphosphonate, S^2−^, benzoate, fluoroacetate, methanesulfonate, or trioctylphosphine [12,76,99,106,107,108,109,110]. One, such example is the usage of sodium dodecyl sulfate as the surface ligand which suppressed the undesirable trap states for higher PLQY (96%), as well as increased the stability of the optically active CsPbI_3_ phase for more than a month, as shown in Figure 6d. The authors found that the advantages were due to the higher binding constant of the SDS ligands than the OA ligands. The groups of Zhang and Pradhan prepared CsPbX_3_ NCs with a low surface V_X_ density by introducing organic halides with large steric hindrance, along with regular ligands [111,112]. The organic halides dictate the reaction but are not passivated on the surface at the end of the reaction [113].

Zwitterionic long-chain molecules are another strategy to stabilize the CsPbI_3_ NCs by tight binding through simultaneous coordination of the surface cations and anions on the NC surface. Even after the synthesis, the final zwitterionic molecule-protected NCs are charged, which is essential for the transport properties. Consequently, the CsPbI_3_ NCs protected by multiple functional groups exhibit higher PLQY rather than the capping agents with single head group. For example, the zwitterionic molecules, including sulfobetaine, phosphocholine, and γ-amino acid, contain both the deprotonated acid group and the quaternary ammonium in order to tightly bound to the CsPbI_3_ by the chelate effect [114,115]. There are also multi-functional ligands, including both phosphate and ammonium cation to effectively passivate the MA^+^, Pb^2+^, and I^−^ defects [116]. There are several ligands (linear OA, squaraine, polyaniline, and quaternary ammonium salts, monoammonium ZnP, and spherical molecular ligands) used successfully to stabilize the bulk MAPbI_3_ films but that are not successful with the CsPbI_3_ NCs. This is likely because the NCs carry a large surface curvature [117,118,119].

### 9.2. Post-Synthetic Passivation of CsPbI_3_ NCs

The main purpose of the post-synthetic passivation of the symmetric CsPbI_3_ NCs, and the role of passivation, is mainly to fill the V_X_ defects on the surface, as negligible effects from the lead vacancy (V_Pb_). Typically, Cs^+^ defects could be minimized by being replaced with ligands containing ammonium cation, while maintaining its interaction with next neighbor I^−^ through hydrogen bonding [120]. The V_X_ defects could be replaced by the ligands possessing a basic nature (soft Lewis bases), and the head group sizes are well-matching halide ions. Liu et al. used ammonium halide as the precursor to constructing halide-rich NCs [121]. During and after the growth, the excess halide ions in the solution can fill the surface vacancy efficiently, contributing to reducing the nonradiative process and consequently enhancing the PLQY. A neutral molecule, such as pyridine or thiophene, are best suited to coordinate to lead (a weak acid). For the improved stability of CsPbI_3_ NCs, Tian et al. used short aminothiols as a substitute ligand for the partial replacement of the long-chain ligands [122,123]. In parallel, passivating the I^−^ ion vacancies on the surface of the α-CsPbI_3_ perovskite, and to obtain a monoexponential PL lifetime and a better PLQY, the following strategies is frequently used. i.e., adding SCN^−^, F^−^, AcO^−^ anions, aromatic amine, and tetraoctylammonium bromide reagents into the NC solution directly at RT, to replace the long-chain ligands [124,125,126,127,128,129,130]. A few other salts, including tetrafluoroborate and ZnX_2_, are used as the post-treating agents to enhance the PLQY of the CsPbI_3_ NCs [131,132] to close to 100%. It is also known in the case of CsPbI_3_ NCs, that they lose their “red” PL emission with time, as a result of releasing the ligand from the NC surface. In such situation, Wang et al. showed an interesting PL recovery approach on the CsPbI_3_ NCs [100]. Initially, the 15-day aged CsPbI_3_ NCs with poor PL emission were taken as the starting material (Figure 6e). As shown in Figure 6e, the recovery of the PL emission was observed after the addition of a tiny amount of ligand, trioctylphosphine (TOP), however the in-depth understanding of such a recovery is not clear. In another report, Yang et al. prepared a rich potassium bromide surface on CsPbBr_x_I_3−x_ NCs through the usage of potassium-oleate in the reaction. This passivation has many advantages, such as inhibiting halide segregation, and suppressing V_X_ defects, and the NCs being stable and highly efficient in the pure red region (637 nm) [133].

**Figure 7:**
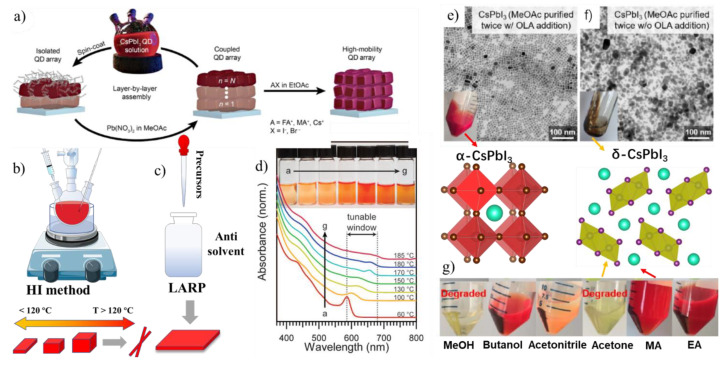
(**a**) Schematic representation of CsPbI_3_ NCs film deposition process with MeOAc and AX salt post-treatment. The figure adapted with permission from [38]; Copyright 2017 AAAS. (**b**,**c**) schematic of the hot injection and LARP methods; (**d**) UV-visible absorption spectra of quantum confined CsPbI_3_ NCs synthesized at different temperatures. The figure adapted with permission from [71]; Copyright 2016 AAAS. (**e**–**g**) are photographs and TEM images to display the influence of washing procedures while sedimenting the CsPbI_3_ NCs with the number of washing times and type of solvents used. The figure adapted with permission from [137], Copyright 2018 American Chemical Society.

Figure 7a shows the ways to improve the potentiality of the CsPbI_3_NCs in solar cells. The bulky ligands should be replaced with short-chain ligands (short-chain acetate) via a solid-state ligand exchange, for improving the charge transport among the NCs which leads to enhanced PCEs in the solar cells [38,71,134,135]. However, the resultant thin films are susceptible to moisture penetration in the absence of a hydrophobic layer, due to the removal of long chain hydrophobic ligands [38]. Hence, the FAI post-treatment is necessary, as shown in the last step of Figure 7a. This step needs to be completed with extreme care, as it is sensitive to reaction time and during the reaction it might hybridizes the fully inorganic CsPbI_3_ NCs with organic FA cations through A-site exchange, leading to an undesired decrease in E_g_ and, subsequently, drop in the open-circuit voltage of the solar cell [134]. In general, the sequential ligand post-treatment strategy is hard, considering the bipolar sites and the soft mismatch of the CsPbI_3_ NCs. Lan et al. [136] developed a sequential ligand post-treatment with two cationic ligands (HPA^+^ and TBS^+^) that can collectively passivate ~5 nm CsPbI_3_ NCs and efficiently replace part of the long chain ligands without influencing the quantum confinement effect. Moreover, the soft acid TBS^+^ binding tightly to the surface of CsPbI_3_ NCs can significantly enhance the stability of the QDs. Based on the high-quality ~5 nm, the CsPbI_3_ NC films fabricated efficient and color-stable pure red perovskite LEDs, with a peak external quantum efficiency of 6.4% and an electroluminescence emission centered at 630 nm.

The surface modification methods widely used in the hybrid perovskite solar cells have also contributed to the surface improvement of CsPbI_3_ perovskites (e.g., phenylethylamine, PEAI) as a surface cation). In general, the surface cation termination not only improves the device properties but also stabilizes a particular crystal phase. Fu et al. have shown that the incorporation of the OLAm cations stabilizes the CsPbI_3_ perovskite in the cubic phase, whereas the use of phenylalkylammonium cation (PEA^+^) additives stabilize the CsPbI_3_ perovskite in the tetragonal phase. Consequently, the PEA-incorporated CsPbI_3_ NC thin films are highly stable under ambient conditions for 15 days, and its solar cell device performance has an efficient PCE of 14.1%. Recently, Dai et al. demonstrated that the “functional conjugated ligands” act as efficient charge transport agents in comparison to the long chain alkyl ligands [138]. The post-synthesis replacement of OA and OLAm ligands by p-mercaptopyridine, iminodibenzoic acid, and PEA cations leads to improved charge transports and device stability [107,139,140,141,142,143]. Table 1 summarizes the PCEs achieved from CsPbI_3_ NC based PSCs through various fabrication methods.

## 10. Size and Shape-Controlled Synthesis of CsPbI_3_ Nanostructures

The current rapid progress on the CsPbI_3_ perovskites paved the way for large-scale synthetic approaches, with a high PLQY leading them to various device applications (Figure 7b,c). Such quick progress is due to the benefits received from the relatively mature field of conventional colloidal nanocrystals, where excellent atomic-level knowledge is at hand. However, most of the successful synthesis was achieved for the CsPbI_3_ nanocubes in comparison to other morphologies, as a result, these CsPbI_3_ nanocubes have been heavily studied and provide promising results for LEDs, lasers, and solar cells. Because of the low formation energies and ionic nature of Cs-Pb-I system, the majority of the reported synthetic protocols are of single-step bottom-up synthesis approaches, in which all of the metal ion precursors, along with their coordinated ligands, are dispersed in a solvent for a further reaction to take place by applying sufficient energy (heat, light, sonication, etc.). Another good protocol is the ligand-assisted re-precipitation method (LARP) approach (schematically illustrated in Figure 7c), that generally yields either spherical NCs or nanoplatelets (NPls) [144,145]. In 2011, Im et al. used to explore MAPbI_3_ NCs on a TiO_2_ matrix as a potential sensitizer for PVs [146]. In their work, the NCs were synthesized on a nanocrystalline TiO_2_ surface by spin-coating the perovskite precursor solution. Later, in 2015, Protesescu et al. [95] developed a hot-injection (HI) method to synthesize the CsPbX_3_ NCs with the high PLQYs (up to 90%). In this method, the PbX_2_ precursors along with ligands in octadecene, were followed by the injection of Cs-oleate at a high temperature and an inert atmosphere (Figure 7b). Note that no polar solvent was involved in HI method, which is beneficial for the CsPbI_3_ NCs. It is one of the early works to inspire the colloidal NC research community with the aim of better stability and shape control and it is still the most frequently adopted method for diverse reaction conditions. Initially, the CsPbX_3_ NCs were reported to crystallize in the cubic perovskite phase (with undistorted [PbX_6_]^4^^−^ octahedra) [95]. However, later studies using synchrotron-based X-ray diffraction confirmed the presence of octahedral distortion in CsPb(I_x_Br_1−x_)_3_ NCs [147,148]. Regarding the PL emission, the CsPbI_3_ NCs succeeded in achieving a high PLQY up to 100%, while the CsPbCl_3_ NCs suffered from lower PLQYs [95,149,150]. Some of the shapes under the pure synthetic conditions did not work well enough for CsPbI_3_, due to the different surface chemistry. For example, the secondary aliphatic amine ligands produced nearly pure CsPbBr_3_ NCs, but the formed CsPbI_3_ NCs quickly degraded [151].

Typically, the noble metal or conventional QD synthesis is understood based on their growth kinetics. However, the CsPbI_3_ formation in the HI method is purely based on the temperature [152], due to the no time-lag between nucleation and the growth processes (1–3 s). Due to this reason, the size can be tunable based on the temperature; the higher the temperature, the larger the NCs (15–20 nm), while at lower temperatures, the smaller the NCs. Figure 7d shows the temperature-dependent CsPbI_3_ NC and their quantum-confined absorption spectra. In another report, Dong et al. [153] proposed another influencing factor, i.e., the relation between the halide to Pb ratio, where the higher ratio leads to smaller CsPbX_3_ nanocubes being observed. A highly implemented strategy to produce 100% PLQY and stable CsPbI_3_ NCs are the usages of TOP−PbI_2_ as a precursor in the synthesis reported by Liu et al. [154] This is similar to that of the previous HI method, where the pre-synthesized TOP−PbI_2_ precursor was inserted into the Cs-precursor mixture with OA/OLAm in octadecene. Following this report, Wu et al. used a highly branched ligand, namely, trioctylphosphine oxide (TOPO), in the HI method. Interestingly, this method produced highly stable and shape-pure CsPbI_3_ nanocubes that retained their structure even at 260 °C [155]. Most of the HI-based methods for the synthesis of CsPbI_3_ used the PbI_2_ reagents for the Pb and I ion sources [132,156]. This type of precursor approach (Cs: Pb: 2I) cannot fully maintain the stoichiometry ratio in the final composition as available in the perovskites (Cs: Pb: 3I). This approach limits the stability of the NCs during the purification process and often leaves defects on the surfaces. To overcome this issue, Qian et al. reported a three-individual precursor (Pb(CH_3_COO)_2_, NH_4_I, and Cs^+^) approach for the synthesis of the CsPbI_3_ NCs. In this way, one can selectively enhance the precursor amount, for example, by providing the iodide-rich environment that enhanced the reaction yield, as well as reducing the formation of defective NCs [157]. Imran et al. [158] reported another HI-based three precursor approach, where benzoyl iodide, cesium carbonate and lead acetate trihydrate along with the ligands for monodisperse CsPbI_3_ NCs. This three-precursor approach provides flexibility to turn both the cations (Cs^+^ and Pb^2+^) and the halide (X^−^) precursors individually in the synthesis. Interestingly, this method produced nearly monodisperse nanocubes, which seems difficult to obtain using the two precursor-based methods. In 2017, Chen et al. reported the solvothermal synthesis of CsPbI_3_ NCs by heating the precursors at 160 °C, along with the ligands in a Teflon-lined autoclave [159]. In comparison to the many studies on CsPbI_3_ NCs, the synthesis of CsFAPbI_3_, FAPbX_3_, and MAPbI_3_ nanocubes have been rarely reported [160,161]. Krieg et al. [114] reported zwitterionic capping ligands to enhance the stability and durability of the CsPbI_3_ nanocubes. The synthesis is again HI based, and includes the injection of pre-synthesized TOP-I_2_ into a mixture of pre-synthesized Cs and Pb complexes along with zwitterionic ligand at 160 °C. Interestingly, the authors showed that the morphology and optical properties of these NCs were preserved after several washing cycles, as the multi-charged zwitterionic ligand simultaneously binds the surface cations and anions.

Tong et al. [149] and Hintermayr et al. [162] reported the synthesis of perovskite NPls by sonicating the dispersion of the perovskite precursors in the presence of the coordinating ligands. Similarly, Pradhan et al. [163] showed that the post-synthetic aging of colloidal solutions leads to the transformation of CsPb(Br_x_I_1__−x_)_3_ into the corresponding NWs with a length of up to several micrometers. In 2015, Zhang et al. [164] reported the solution phase colloidal synthesis of CsPbBr_3_ perovskite NWs that exhibited an orthorhombic crystal structure. They found that, different to the linear growth mechanism in the hot injection synthesis, the initially formed nanocubes gradually transformed into NWs through the oriented attachment mechanism. Unlike in the case of the colloidal inorganic CsPbBr_3_ perovskite NWs, only limited research progress was made regarding the controlled synthesis of the optically active CsPbI_3_ and MAPbI_3_ nanowires. Most of the studies have used other techniques, such as growing them on templates, and the substrates solution–phase synthesis of high-quality NWs for optoelectronic and photovoltaic applications [165].

**Table 1:** Summary of PSCs fabricated base on CsPbI_3_ NCs and their variants, respective methods to receive the improved performances.

Type of NCs		Device Structure	SE/CEI/Ad/IE	PCE (%)	Year [Ref]
CsPbI_3_		FTO/TiO_2_/NCs/spiro-OMeTAD/MoO_x_/Al	Methyl acetate (SE)	10.77	2016 [71]
CsPbI_3_		FTO/TiO_2_/NCs/spiro-OMeTAD/MoO_x_/Al	AX-coating (SE)	13.43	2017 [38]
CsPbI_3_		FTO/TiO_2_/NCs/PTB7/MoO_x_/Ag	NCs as interface layer (IE)	18.56	2018 [166]
CsPbI_3_		FTO/TiO_2_/NCs/spiro-OMeTAD/Au	Short ligands (SE)	11.87	2019 [104]
CsPbI_3_		FTO/TiO_2_/NCs/PTAA/MoO_3_/Ag	Mercaptopyridine (SE)	14.25	2020 [141]
CsPbI_3_		FTO/Cl@SnO_2_/NCs/P3HT/MoO_x_/Ag	ETL (IE)	14.5	2021 [167]
CsPbI_3_		FTO/TiO_2_/NCs/PTAA/MoOx/Ag	Cs^+^ coating (SE)	14.1	2019 [168]
CsPbI_3_		ITO/SnO_2_/NCs/spiro-OMeTAD/Ag	Amino acid (SE)	13.66	2020 [169]
CsPbI_3_		FTO/c-/s-m-TiO_2_/NCs/spiro-OMeTAD/Au	IE	14.32	2020 [170]
CsPbI_3_		FTO/TiO_2_/NCss/spiro-OMeTAD/Au	GeI_2_ (Ad)	12.15	2019 [171]
CsPbI_3_		FTO/TiO_2_/NCs/spiro-OMeTAD/MoO_x_/Al	A-site (CEI)	13.47	2018 [172]
CsPbI_3_		FTO/TiO_2_/GR-NCs/PTAA/Au	µ-Graphene (Ad)	11.40	2018 [173]
CsPbI_3_		FTO/TiO_2_/NCs/PTAA/MoO_x_/Ag	Amines (SE)	15.0	2020 [174]
CsPbI_3_		FTO/TiO_2_/NCs/PTAA/MoO_x_/Ag	FAI (SE)	13.1	2020 [175]
CsPbI_3_		FTO/TiO_2_/NCs/spiro-OMeTAD/MoO_x_/Al	Zn-doped (CEI)	16.07	2020 [34]
CsPbI_3_		FTO/TiO_2_/NCs/spiro-OMeTAD/Ag	Zn-doped (CEI)	14.8	2020 [176]
CsPbI_3_		FTO/TiO_2_/NCs/PTB7/MoO_3_/Ag	Yb-doped (CEI)	13.12	2019 [39]
CsPbI_3_		FTO/TiO_2_/NCs/spiro-OMeTAD/Au	spray-coated (IE)	11.2	2019 [177]
CsPbI_3_		ITO/TiO_2_/NCs/PTAA/MoO_3_/Ag	Phenylalanine (SE)	14.60	2020 [126]
CsPbI_3_		FTO/TiO_2_/NCs/Spiro-OMeTAD/MoO_x_/Ag	Methyl acetate (SE)	12.85	2021 [178]
CsPbI_3_		FTO/TiO_2_/NCs/pcbm/Spiro-OMeTAD/MoO_x_/Au	phenethylammonium cations (SE)	14.10	2020 [179]
CsPbI_3_		FTO/TiO_2_/NCs/PTAA/MoO_3_/Ag	HNC(NH_2_)_2_-coating (SE)	15.2	2020 [180]
CsPbI_3_		FTO/TiO_2_/NCs/PTAA/MoO_3_/Ag	Electron acceptors (Ad)	15.10	2021 [181]
CsPbBr_1.5_I_1.5_		FTO/TiO_2_/NCs/Spiro-OMeTAD/MoO_x_/Ag	X-site (CEI)	9.70	2021 [182]
CsPbI_3_	Rigid	ITO/SnO_2_/PCBM/NCs/PTB7/MoO_3_/Ag	IE	15.1	2021 [183]
	Flexible		IE	12.3	
CsPbI_3_		ITO/SnO_2_/NCs/Spiro-OMeTAD/Ag	X-supply (SE)	16.20	2021 [184]
CsPbI_3_		FTO/TiO_2_/NCs/PTAA/MoO_3_/Ag	Organic dopant to NCs (IE)	12.30	2021 [185]
CsFAPbI_3_		ITO/TiO_2_/NCs/spiro-OMeTAD/MoO_x_/Al	Layer by layer deposition (IE)	17.4	2019 [186]
CsPbBr_3_		FTO/ZnO/QDs/spiro-OMeTAD/Au	—	6.81	2018 [187]
CsPbBr_3_		FTO/TiO_2_/NCs/Spiro-OMeTAD/MoO_x_/Ag	—	4.20	2020 [188]
CsPbBrI_2_		ITO/TiO_2_/QDs/P3HT/Au	X-site (CEI)	12.2	2020 [189]
CsPbI_2+x_Br_1−x_		FTO/TiO_2_/QDs/PTAA/Au	X-site (CEI)	14.45	2018 [190]

The abbreviations “SE” refers to surface engineering via ligands, inorganic complexes, organic molecules; “IE” refers to interface engineering through chemical process, electron transport layer (ETL), etc.; CEI refers to crystal engineering through ion exchange (A, and X- sites); “Ad” refers to the additives introduced to increase the charge transfer.

### Purification Techniques of CsPbI_3_ NCs for Ready to Use in Solar Cells

Most of the synthesis procedures are conducted at higher molar amounts of ligands. Without subjecting them to washing procedures, the presence of the excess ligands could degrade the CsPbI_3_ to its non-perovskite form. For many of the applications, such as solar cells or light-emitting diodes that require charge transport through a deposited layer of NCs, the presence of the excessive ligands, diminishes or confuses the results. Typically, the used antisolvents should be anhydrous for stabilizing the final CsPbI_3_ sediment for longer times. Glove box conditions are commonly employed for handling these NCs for sensitive experiments [7]. While precipitating the NCs, one precipitative washing step is not enough to achieve the necessary level of purity required for the analytical studies. At least two cycles of precipitative washings are needed [96]. A second precipitative washing step with antisolvent can degrade these CsPbI_3_ NCs. A well-accepted procedure to purify the NCs from their solution is by subjecting to sedimentation by mixing the antisolvent followed by centrifugation. The early research of CsPbI_3_ NCs purification commonly used ethyl acetate or a tert-butanol (BuOH) treatment. Swarnkar et al. found that the weak acid-base interaction of I^−^ and oleylammonium, results the removal of surface protected ligands along with the unbound ligands, which can cause irreversible agglomeration following an undesired phase transformation and a significant drop in photoluminescence [71].For example, typically, the α-CsPbI_3_ nanocubes transform to the δ-CsPbI_3_ [67], and the MAPbI_3_ nanocubes decompose into PbI_2_ [137].

The factors affecting the purification process are anti-solvent polarity, the number of washing times, the volume ratio of antisolvent to solvent, and the time of centrifugation. The NC degradation could be minimized by direct centrifugation in the absence of polar antisolvent or by selecting a suitable solvent with compatible polarity. Direct sedimentation without using any antisolvent is possible by centrifugation at high speeds, but is limited to the larger-sized CsPbI_3_ and Cs_4_PbI_6_ NCs [7,191]. However, the precipitate still leaves the residual amount of unbound ligand, and the low volatility reaction solvent (i.e., octadecene) is also retained in the precipitate of CsPbI_3_ NCs. It also creates challenges during characterization for surface analysis techniques (e.g., TEM, NMR, FTIR, etc.). The longer centrifugation times cause the agglomeration of NCs through crystal fusing which cancauseunwanted results, yielding CsPbI_3_ nanocubes with a very poor dispersibility, low PLQYs, and nanocubes largely transformed to the yellow phase [94,192]. The precipitation of the CsPbI_3_ and MAPbI_3_ NCs with methanol and acetone turned the color of the precipitate into pale yellow or milky white. The methanol and acetone had less solubility in octadecene and are not compatible with CsPbI_3_ and MAPbI_3_ NCs [7]. Thus, such polar solvents should be avoided when precipitating the CsPbI_3_ and MAPbI_3_, and the FAPbI_3_. On the face of it, the purification of the CsPbI_3_ NPls and CsPbI_3_ NWs without structural modification is quite challenging. Recently, Swarnkar et al. reported a well-controlled purification method for CsPbI_3_ NCs through methyl acetate treatment; this not only improved the yield of the reaction but also improved the device performance. The acetate ions in the methyl acetate exchanged with the oleate ligands on the surface of NCs, resulting in improved charge transport properties. Figure 7e shows the images of CsPbI_3_ NCs, that were sedimented with various solvents traditionally used for NC purifications, Among them, the solvent methyl acetate (MA) sediments the CsPbI_3_ NCs effectively by transparent supernatant and the red colored nanocubes around the centrifuge tube. Other solvents, such as ethyl acetate, could not sediment the NCs completely, thus reducing the yield. To prevent degradation, a small amount of the excess ligand (i.e., OLAm) must be added before the second precipitative wash [137]. Figure 7e,f shows the TEM images and photographs of the CsPbI_3_ nanocubes upon a two-time wash with MA solvent in the presence and absence of minute amounts of additional OLAm. It is clearly seen that in the presence of OLAm, the CsPbI_3_ NCs do not lose their surface passivated ligands and the shape remain intact. In the case of the direct washing two times with MA, this led to a faint-colored precipitate due to the presence of the partially degraded “ill-defined” CsPbI_3_ NCs with very poor emission properties and no further redispersion in a solvent. In addition, these particles tended to decompose quickly to yellow CsPbI_3_. In general, to obtain the NCs free from the additional ligands, one needs to optimize the anti-solvent precipitate methods depending on the reaction conditions (size of NCs, ligands, and concentration).

## 11. Conclusions and Outlook

The greater attention paid to CsPbI_3_ NCs is due to their ideal band gap for efficient solar cell applications; they are especially preferred as the top cell in tandem solar cells. Interestingly, their complete inorganic nature offers relatively higher stability than the hybrid perovskites (e.g., MAPbI_3_) and they are emerging as study material for various branches of science and engineering. Therefore, the efforts continue to improve their efficiency, stability and overall, their applications in the solar cell. The key issue for achieving efficient devices based on CsPbI_3_ is to resist the phase transformation of the optically active structure to non-perovskite forms during the device working conditions. Various factors affect the bandgaps, due to the soft nature of these perovskites and the dynamic tilting of the octahedra within the perovskite lattice, resulting in observation of various CsPbI_3_ polymorphs (α, β, γ, and δ). This field is mature in terms of the fundamental understanding of the crystal phases, surface defects, and crystal engineering. In this review, we first focused on the fundamental aspects of the CsPbI_3_ crystal phases, the factors to decide their stability (tolerance factor and octahedral factor), and the structural relationship among CsPbI_3_ the polymorphs, and the factors affecting the bandgaps, surface defects, traps, and defect-tolerance structure. Size engineering is a great leap to shift the thermodynamic stability at room temperature from δ-CsPbI_3_ to optically active phases, α, β, and γ-CsPbI_3_. Even though the CsPbI_3_ is fortunate to have a natural defect tolerancestructure as a result of band gap opened within the antibonding orbitals, the excessive surface that traps mainly halide vacancies damages this property and reduces the photoluminescence of the NCs.

In order to achieve higher solar efficiencies, it is important to enhance the charge mobility among the components, i.e., within the CsPbI_3_ grain boundaries or CsPbI_3_ NCs array, as well as at the interfaces between the absorber and the charge transport layers. Research is going in this direction via the surface molecular joints or the incorporation of additives (e.g., metal halides and graphene). In the second part of the review, we mainly focused on the surface chemistry, and the methods to minimize the surface traps were discussed in detail. They include the in-situ synthesis methods and the post-treatment methods (dicarboxylic acid, multifunctional head ligands, zwitterion molecules, polymers, ZnI_2_, etc.) for near 100% PLQY with cubic-phase stability has been achieved. The recent progress in crystal engineering through ion exchange (Cs^+^, Pb^2+^, and X^−^) methods offer far-reaching promises and expectations for these CsPbI_3_ NCs. At optimal amounts of the exchange process, the obtained alloy, CsPbI_3_, offers greater stability, with a PLQY reaching above 90%.

The crystal engineering in the CsPbI_3_ NCs through these ion exchange processes in CsPbI_3_ NCs promise greater hope than ever before, for the futuristic solar cells. For example, the Cs^+^ exchange with FA increases the charge mobility eventually for better PSCs. In another example, the Pb^2+^ exchange with the smaller cations (Mn^2+^, Sr^2+^, Sn^2+^ Zn^2+^ and Ni^2+^) enhances the tolerance factor which substantially increases the photostability of a solar device. The detailed mechanistic investigations of the ion exchange process in CsPbI_3_, and the chemical reaction procedures between water and oxygen, need to be further explored. In the case of hybrid perovskites, the large-area scalable fabrication of devices is achieved, however, such large-scale fabrications are rare in the case of the CsPbI_3_ films. Moreover, the currently applied laboratory methods have to be updated by implementing industrial fabrication methods for the commercialization of CsPbI_3_ solar cells. Thus, there is long way ahead to achieve and implement industrial-scale fabrication processes for efficient and stable CsPbI_3_ PSCs.
